# What capacity exists to provide essential inpatient care to small and sick newborns in a high mortality urban setting? - A cross-sectional study in Nairobi City County, Kenya

**DOI:** 10.1371/journal.pone.0196585

**Published:** 2018-04-27

**Authors:** Georgina A. V. Murphy, David Gathara, Nancy Abuya, Jacintah Mwachiro, Sam Ochola, Robert Ayisi, Mike English, on behalf of the Health Services that Deliver for Newborns Expert Group

**Affiliations:** 1 Centre for Tropical Medicine and Global Health, Nuffield Department of Medicine, University of Oxford, Oxford, United Kingdom; 2 Kenya Medical Research Institute/Wellcome Trust Research Programme, Nairobi, Kenya; 3 Nairobi City County Government, Nairobi, Kenya; BRAC, BANGLADESH

## Abstract

**Introduction:**

Appropriate demand for, and supply of, high quality essential neonatal care is key to improving newborn survival but evaluating such provision has received limited attention in low- and middle-income countries. Moreover, specific local data are needed to support healthcare planning for this vulnerable population.

**Methods:**

We conducted health facility assessments between July 2015-April 2016, with retrospective review of admission events between 1^st^ July 2014 and 30^th^ June 2015, and used estimates of population-based incidence of neonatal conditions in Nairobi to explore access and evaluate readiness of public, private not-for-profit (mission), and private-for-profit (private) sector facilities providing 24/7 inpatient neonatal care in Nairobi City County.

**Results:**

In total, 33 (4 public, 6 mission, and 23 private) facilities providing 24/7 inpatient neonatal care in Nairobi City County were identified, 31 were studied in detail. Four public sector facilities, including the only three facilities in which services were free, accounted for 71% (8,630/12,202) of all neonatal admissions. Large facilities (>900 annual admissions) with adequate infrastructure tended to have high bed occupancy (over 100% in two facilities), high mortality (15%), and high patient to nurse ratios (7–15 patients per nurse). Twenty-one smaller, predominantly private, facilities were judged insufficiently resourced to provide adequate care. In many of these, nurses provided newborn and maternity care simultaneously using resources shared across settings, newborn care experience was likely to be limited (<50 cases per year), there was often no resident clinician, and sick babies were often referred onwards. Results suggest 44% (9,764/21,966) of Nairobi’s small and sick newborns may not access any of the identified facilities and a further 9% (2,026/21,966) access facilities judged to be inadequately equipped.

**Conclusion:**

Over 50% of Nairobi’s sick newborns may not access a facility with adequate resources to provide essential care. A very high proportion of care accessed is provided by four public and one low cost mission facility; these face major challenges of high patient acuity (high mortality), high patient to nurse ratios, and often overcrowding. Reducing high neonatal mortality in this urban, predominantly poor, population will require effective long-term, multi-sectoral planning and investment.

## Introduction

Despite considerable progress in reducing child mortality globally, slower progress has been made in neonatal survival. Consequently, neonatal mortality now accounts for over 40% of all child mortality in many low- and middle-income countries (LMICs).[1] Much of this is preventable with the provision of universal access to basic, but high quality, health services.[2–4] While international and national policies recognise the need to create demand for, and supply of, skilled delivery and maternal health facilities, rather less attention has been paid to the provision of high quality care for sick newborns in LMICs.[5–7] Furthermore, efforts to measure service provision and quality in LMICs[8, 9] generally do not provide insight into the percentage of the population not accessing care (underuse) and often focus solely on public sector facilities.[10] [5, 11]

In Kenya, it is estimated that efforts to deploy effective interventions averted 10,500 neonatal deaths between 2003 and 2014, with optimal care and management during birth and the postnatal period accounting for 70% of all deaths averted.[12] In Nairobi City County, Kenya, 60–70% of the population live in slums and income inequality is high.[13] An estimated 88.7% of births take place within health facilities, compared to 61.2% on a national level.[14] Yet, the neonatal mortality rate in Nairobi is considerably higher than elsewhere in Kenya (39 compared with 19–25 per 1,000 live births respectively).[14] Data are needed to understand the gaps in provision, access, and quality of health services that may explain this high mortality rate among newborns. Such data could also inform improved, targeted, and evidence-based local policy on equitable and quality healthcare provision, particularly for low income groups.

This study aims to describe the provision of and access to inpatient neonatal services within the local pluralistic health-care system of Nairobi City County, a large high-mortality urban population. The gap between the number of small and sick newborns needing and access care is determined; and the structural capacity of facilities to provide quality care is examined.

## Methods

We conducted a cross-sectional study during July 2015-April 2016, including health facility assessment and retrospective review of admission events between 1^st^ July 2014 and 30^th^ June 2015, of health facilities providing inpatient neonatal care in Nairobi City County as part of the Nairobi Newborn Study. Full details of the study protocol have been published elsewhere.[15] The full study manual, data collection tools, and standard operating procedures have also been made publicly available.[16]

### Identification of health facilities

**Fig 1:**
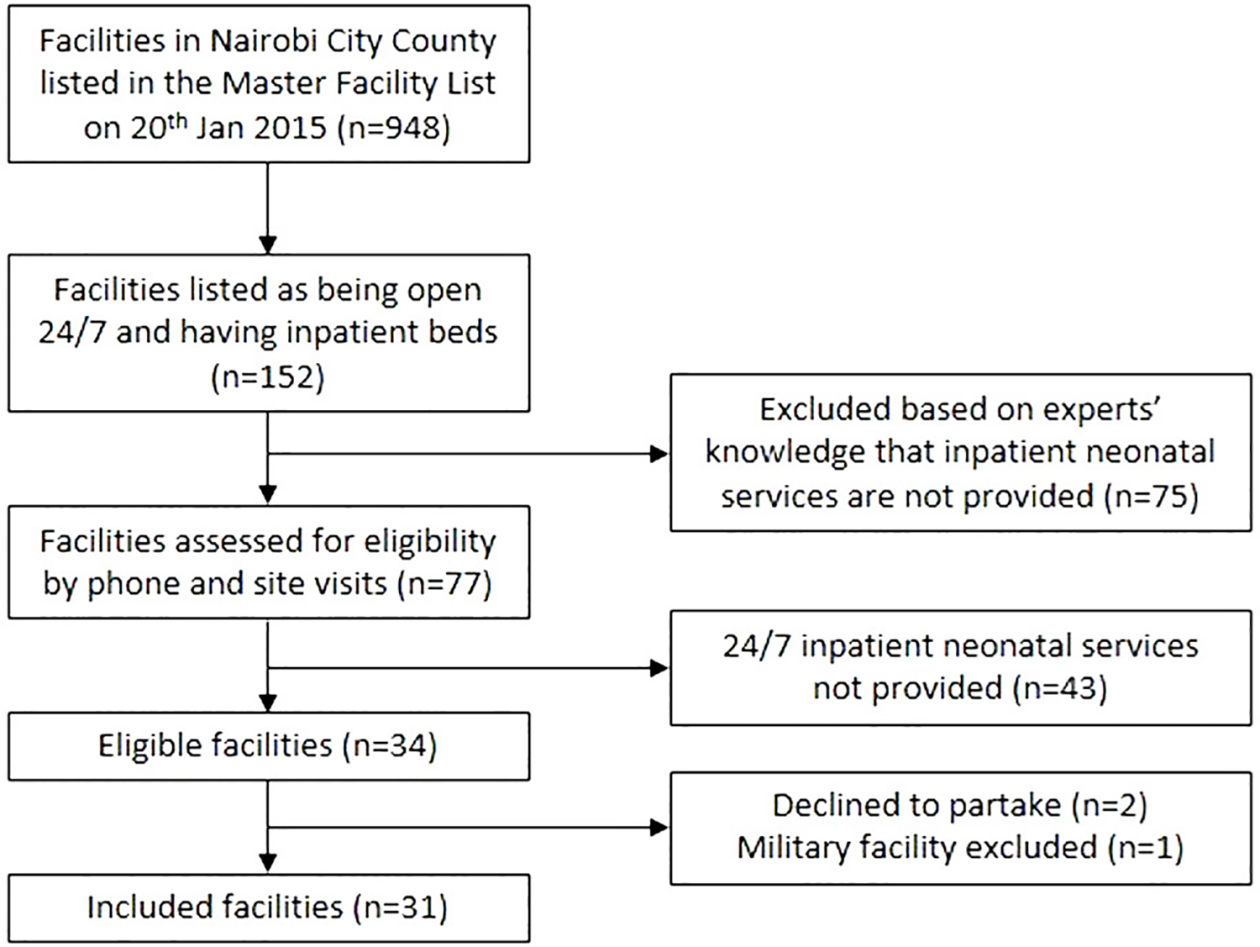
STROBE study flow chart for study facility selection.

We defined inpatient neonatal care (INC) facilities, the subject of the study, as those providing inpatient care for sick newborns 24 hours a day for seven days a week (24/7). This definition focuses on facilities aiming to offer continuous care for a sick newborn and excludes those that focus on providing care to women with uncomplicated deliveries and normal newborns that operate a ‘treat and transfer’ policy if they identify a sick newborn (or mother). INC facilities from all sectors in Nairobi City County–public, not-for-profit private (mission), and for-profit private (private)–were identified using a step-wise approach. First, the Kenya Master Facility List was consulted to identify facilities listed as hospitals and/or open 24/7.[17, 18] Second, expert opinion from local obstetricians and paediatricians was used to exclude facilities not meeting the 24/7 criteria and add facilities that may be eligible and missing from the Master Facility List. Thirdly, all potential facilities were telephoned to evaluate their eligibility and then visited by the research team to confirm eligibility. Lastly, during these visits, advice was sought from facility staff on additional facilities that may have been missed from our list. Fig 1 shows the STROBE study flow chart for facility selection.

### Resource indicators (structure)

An assessment was conducted in facilities that agreed to participate by clinically trained research team members using a paper-based, structured survey tool to examine staffing, infrastructure, and equipment and consumables supporting care in the maternity and newborn units (NBUs). Information on staffing and costing was collected through interview with the nurse(s) in charge of the maternity unit and NBU, and later confirmed through telephone follow-up. Information on infrastructure and equipment/drugs was collected through direct observation.

Staffing numbers reflect the staff on duty during a typical weekday day-time shift. Facilities were considered to have dedicated NBU nurses if their NBU was staffed by nurses who did not have responsibilities for other patient groups or other wards, such as maternity or general paediatrics, during their shift; hence, dedicated nurses’ time was spent caring for small and sick newborns only.

Equipment was considered to be available if it was both on the ward and working on the day of the visit. Items available on either the maternity ward or NBU that were described by facility staff as being shared between the two locations, were considered to be available for both maternity and the NBU (e.g. a suction machine). Drugs were considered to be available if they were on the ward or accessible within five minutes without administrative barriers (such as prepayment or waiting for keys from a senior hospital staff member not present on the ward). A drug was considered to be ‘in store’ if it was available within the facility and within 2 hours of request but not available on the ward (by the above criteria).

### Clinical care (process) and outcomes

Admission data on patient characteristics and outcomes were collected from facility registers for all neonatal admissions to each facility during the period 1^st^ July 2014 and 30^th^ June 2015. Where neonatal admission registers were not available or admissions were fewer than 20, the admission information was obtained from individual patient medical records. We considered admissions only requiring supportive care (e.g. observation of a baby after a mother’s caesarean) or for only a minor condition (e.g. caput succedaneum) to be ‘inappropriate admissions’ as care for such conditions can be offered on a suitably staffed postnatal ward and as our interest was in sick newborns requiring interventions beyond simple observation.

### Data entry

Data on admissions were entered onsite in facilities by trained clerks into a purpose-designed standardised data capture tool created in REDCap[19] with inbuilt range and validity checks. Pre-designed cleaning scripts were run daily and weekly on aggregate data and corrections made where possible by referring back to the source documents. Data from the paper-based structural assessment were doubled entered into a REDCap tool by two independent data supervisors. Discrepancies were resolved by reassessing the paper form and discussion with the principle investigator when necessary.

### Calculations and analysis

Data analysis was conducted using Stata version 13 (Stata Corporation, Texas, USA).

Live births for the year mid-2014/mid-2015 were estimated as 120,032 by applying the Nairobi City County crude birth rate (3.1%) obtained from the Kenyan 2014 demographic and health survey[14] to population estimates for the County, derived from the 2009 national census and adjusted for population growth at a rate of 3.89% per year.[20, 21] It was previously estimated that 18.3 per 1,000 live births would require inpatient services in Nairobi City County.[22] We applied this rate of 18.3 per 1,000 live births to the total birth cohort (120,032) to estimate the number of sick newborns requiring care during the study period of 1^st^ July 2014–30^th^ June 2015.

We calculated the total number of neonatal inpatient days for sick newborns (imputing the average length of stay (LOS) where data were missing) and used this to calculate bed occupancy [(total number of patient days during the study period x 100) / (available cots and incubators x 365)][23] and a nurse to patient ratio for a weekday, daytime shift.

A total of 126 items from the structural assessment were assessed across eight domains: i) infrastructure (3 items), ii) laboratory services (10 items), iii) hygiene equipment (14 items), iv) safe delivery equipment and drugs for mothers (37 items), v) resuscitation equipment for newborns on the delivery ward (20 items), vi) essential equipment in the NBU (18 items), vii) IV fluids and feeds in the NBU (8 items), and viii) NBU drugs (17 items) (listed in S1 and S2 Tables). The 10 item score for laboratory services was made up of 1 point for having all items considered part of a minimum package of laboratory services provided 24/7 (5 tests) (S2 Table). Facilities not able to provide this minimum set of tests scored 0/10 irrespective of other tests they might offer. Then for 9 pre-specified desirable tests offered beyond this minimum package one further point was added (to achieve a maximum of 10) (S2 Table). An overall structure score was calculated as a percentage from the sum of the eight domain scores divided by the total possible score and a simple categorisation of deciles was created.

Facilities were considered to provide ‘inadequate services’ if their structural score was <80%, annual neonatal admissions were <50 per year (deemed an inadequate number of admissions to maintain high level skills), or they did not have any dedicated NBU nursing staff.

### Ethics and permission

Ethical approval was granted by the Kenya Medical Research Institute (KEMRI) Scientific and Ethics Review Unit (protocol No. 2999). Written informed consent to conduct this study was obtained from the Medical Supervisor or equivalent authority in charge of each facility.

## Results

### Availability of inpatient neonatal services

**Fig 2:**
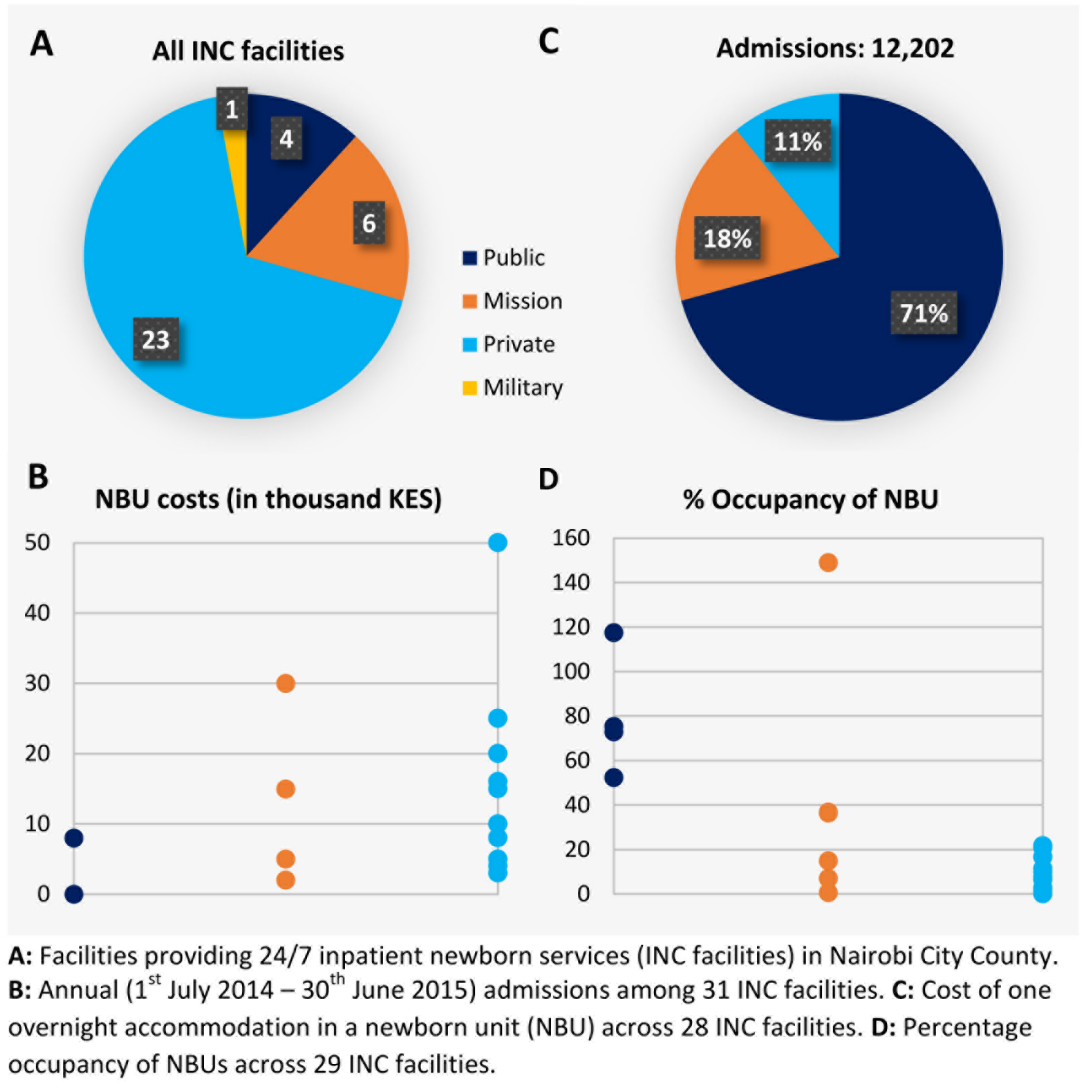
Inpatient neonatal care (INC) facilities in Nairobi City County.

Thirty four INC facilities were found in Nairobi City County: 23/34 were private, 6/34 were mission, and 4/34 were public sector facilities (including a national referral and teaching hospital) (Fig 2A). One of 34 facilities was run by the military and, as only military families could access services, it was excluded from all subsequent analyses. The two small private facilities that declined to partake in the study were estimated to have 250–350 maternal deliveries[24] and <50 neonatal admissions each per year. We, therefore, report newborn admissions and the structural assessment for 31 INC facilities. For 1/31 facilities total newborn admissions data were not available from any source and were estimated by hospital management.

In three public facilities, care was free while in the fourth approximately 8,000 KES (80 USD) per night stay was charged to families at discharge unless the family had a National Hospital Insurance Fund card (32% of Nairobi population[25]). All non-public facilities levied charges ranging from 2,000–30,000 KES (20–300 USD) and 3,000–50,000 KES (30–500 USD) per night stay in the mission and private sectors, respectively (Fig 2B).

### Neonatal admission populations

After removal of inappropriate admissions (n = 2,605), the total number of sick newborn admissions between 1^st^ July 2014 and 30^th^ June 2015 in 31 participating INC facilities was 12,202 (Fig 2B). Fifteen facilities had <50 admissions between 1^st^ July 2014 and 30^th^ June 2015 (small); four had 50–100 admissions (medium); eight had 101–900 admissions (large); four had >900 admissions (very large). Just five facilities accounted for 84% of all admissions; these are three public (accounting for 8,344 admissions), one mission (1,324 admissions, cost = 2,000 KES per night [20 USD]), and one private (632 admissions, cost = 16,000–37,000 KES per night [160–370 USD]) sector facility.

Among small and sick newborn patients with a recorded diagnosis (n = 11,414), the most common admission conditions were birth asphyxia (30.3%), neonatal respiratory distress (26.6%), prematurity (25%), jaundice (10.0%), and severe infection (9.6%); 23.2% (2,649/11,414) newborns had two or more conditions documented. Most patients were admitted within the first 24 hours of life and babies <2000g birthweight (all required to receive kangaroo mother care (KMC) as part of current Kenyan policy [22]) account for at least 15% of admissions (**i** S4 Table).

### Structural capacity

Differences in the organisation of care were observed, with 12 facilities having a discrete stand-alone NBU and 19 providing care for sick newborns in a room or ward area within the maternity ward. Facilities with a greater numbers of neonatal admissions were more likely to have a discrete NBU.

**Fig 3:**
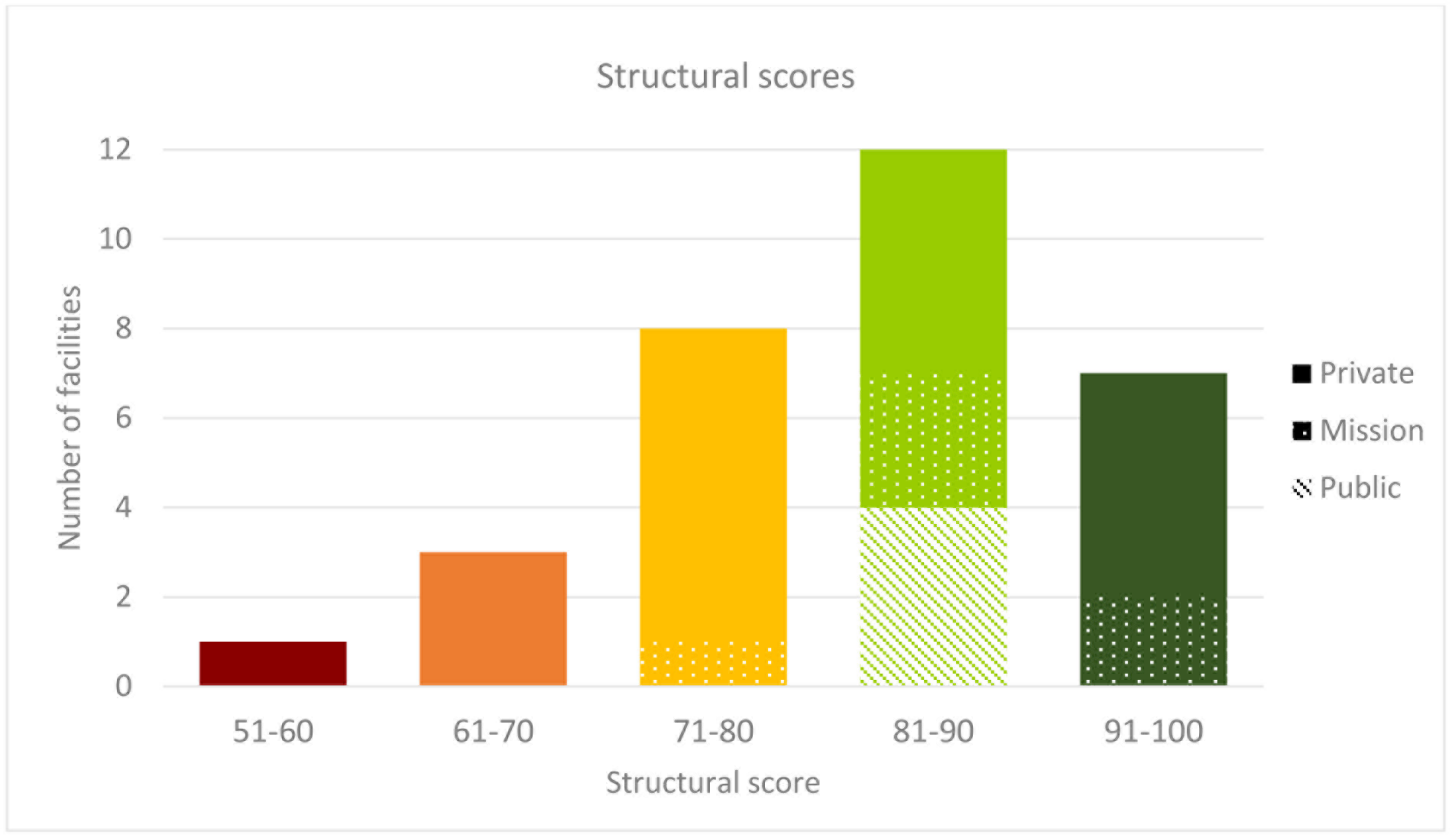
Distribution of structural quality across inpatient neonatal care facilities in Nairobi City County.

All facilities achieved a structural score of over 50%; 19/31 scored over 80%; 7/31 scored over 90% (Fig 3). All four public facilities scored 81–90. Larger facilities tended to have higher scores than smaller facilities (S1 Fig). Safe delivery equipment and drugs for mothers on the delivery ward was the weakest domain (average availability score of 67.8% in all sectors [public score = 80.4%; mission = 78.8%; private = 62.0%]) (S1 Table; details on commonly missing items are presented in S2 Table). Items missing from more than a third of INC facilities’ NBUs included blood transfusion giving set, term formula feed, vitamin K, nevirapine solution, phenobarbitone (intravenous), and penicillin (intravenous) (S2 Table).

Equipment was often shared between the NBU and delivery ward, for example thermometers, weighing scales and suction machines were available in 26/31, 30/31 and 26/31 facilities but shared in 7/26, 9/30 and 7/26 of these facilities. Sharing was more common in facilities where care was provided for sick newborns as part of the maternity ward; however, sharing also occurred in facilities with discrete NBUs. Sharing did not occur in facilities with >100 neonatal admissions.

Of the 17 NBU drugs assessed in facilities and found to be available, a median of 88.9% (range: 5.3–100%) were not immediately available but only available in stores in the private sector hospitals; this practice was less common in mission sector facilities (67.7%; range 18.8–89.5%) and substantially less common in public sector facilities (10.7%; range 0–44.4%).

Items suggesting provision of more advanced care, not included in the structural score, were considered separately. These included lumber puncture needles (available in 6 of 31 facilities), pulse oximeters (24 of 31), ventilators (8 of 31), bubble CPAP (8 of 31), exchange transfusion set/pack (5 of 31), and surfactant (18 of 31). Five facilities (representing 4,053 [33%] admissions) could provide both CPAP and surfactant (available either on the ward or in store).

### Occupancy and staff availability

**Fig 4:**
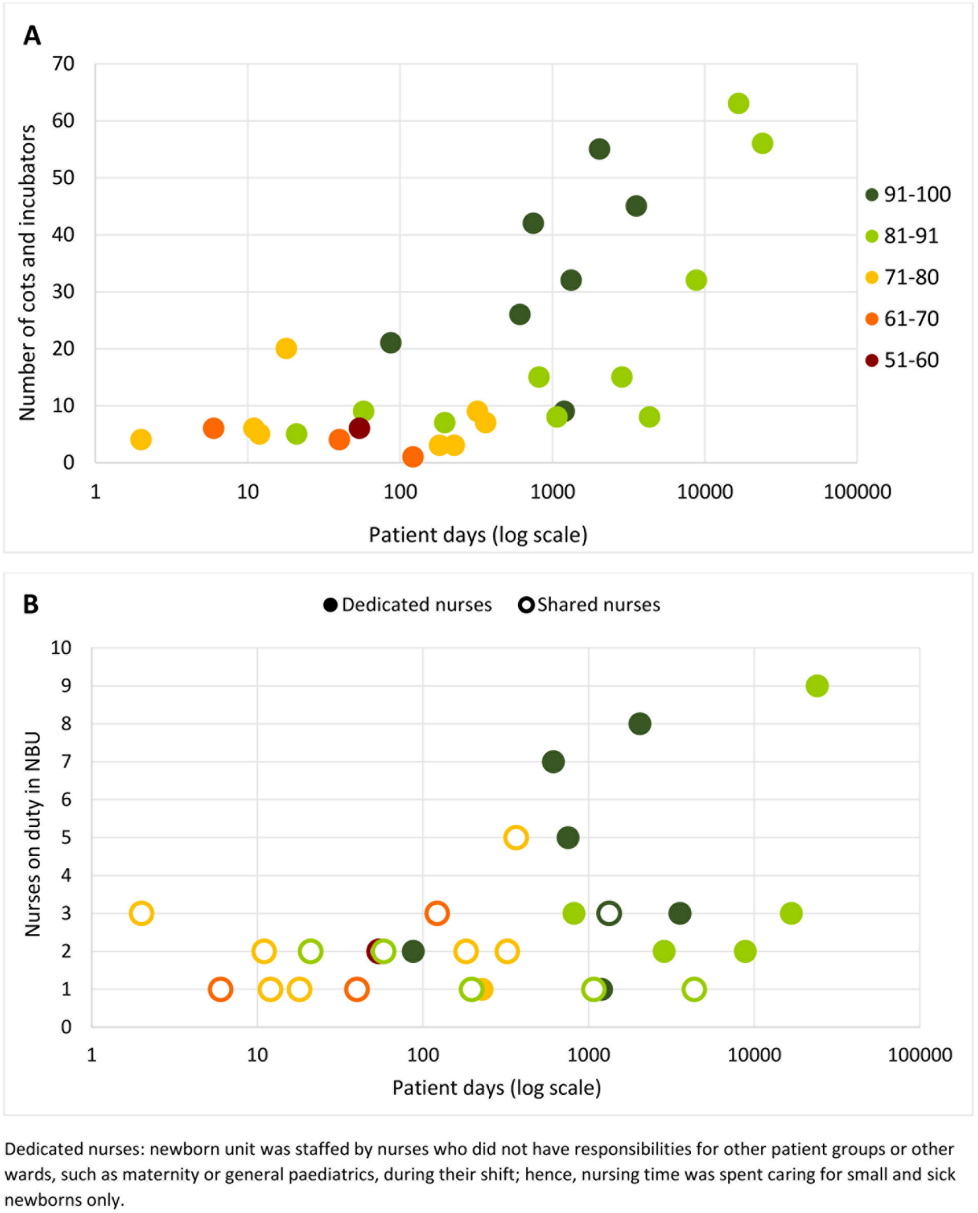
Bed capacity for small and sick newborns (A) and nursing staffing on duty during the day of a weekday on the newborn unit (B) by number of patient days, 1st July 2014–30th June 2015.

A log-linear relationship was found between the total number of inpatient days for a facility (on log scale) and the number of cots and incubators available for newborn inpatients (Fig 4a). Of the 29 facilities with occupancy data, half of facilities (12 private and two mission sector) had occupancy of 10% or less and nine facilities had occupancy of 10–50%. Two facilities, a public and a low-cost mission sector facility, had occupancy greater than 100% (117% and 149%). Occupancy varied greatly between and within sectors (Fig 2d). Incubator sharing (more than one neonatal patient simultaneously using a single incubator) was reported by nine facilities; these included all five facilities with occupancy 80% and greater.

Medical staff data were available from 29 facilities. All facilities reported having at least one paediatrician (range 1–12) and obstetrician (range 1–23) to consult (consultants are non-resident in Kenya). In 7/29 facilities (all private) the consultant managed the neonatal unit supported only by nurses as no medical or clinical officers (non-physician clinicians) were assigned duties covering neonatal care; a further three facilities (all private) had clinical officers but no medical officers covering the newborn unit.

Twelve facilities had dedicated nursing staff for neonatal patients; whereas, for 19 facilities nurses shared their time between sick newborns and patients on the maternity or other wards during their shift. The median number of neonatal patients per nurse was 2.0 (range 0.1–15.3) across facilities with dedicated nursing staff, compared with 0.1 (range: 0.02–11.9) among facilities with shared nursing staff. The number of nurses were fewer in many facilities during the night shifts (typically 7pm to 7am). There was little increase in the number of nurses on duty during a typical day shift as the number of patient days increased (Fig 4b). Among the four public facilities the ratio ranged from 4–15 patients per nurse and this ratio ranged from 3–15 patients per nurse in the five largest facilities. These facilities also reported higher mortality although this may reflect higher acuity (described below).

### Neonatal patient outcomes

No information on patient outcomes was recorded in registers for 21.4% (2,610/12,202) of patients (22.1% in public, 15.4% in mission, and 26.8% in private). Among those patients with a documented outcome: 80.1% (7,680/9,592) were discharged home alive; 13.5% (1,296/9592) died; 6.3% (600/9,592) were referred to another health facility; and 0.2% (16/9,592) absconded. Mortality among the five largest facilities was 14.5% compared to 7.7% among the smaller facilities. Mortality was highest in the public sector (16.5%) compared with the mission (5.9%) and private (7.3%) sectors.

**Fig 5:**
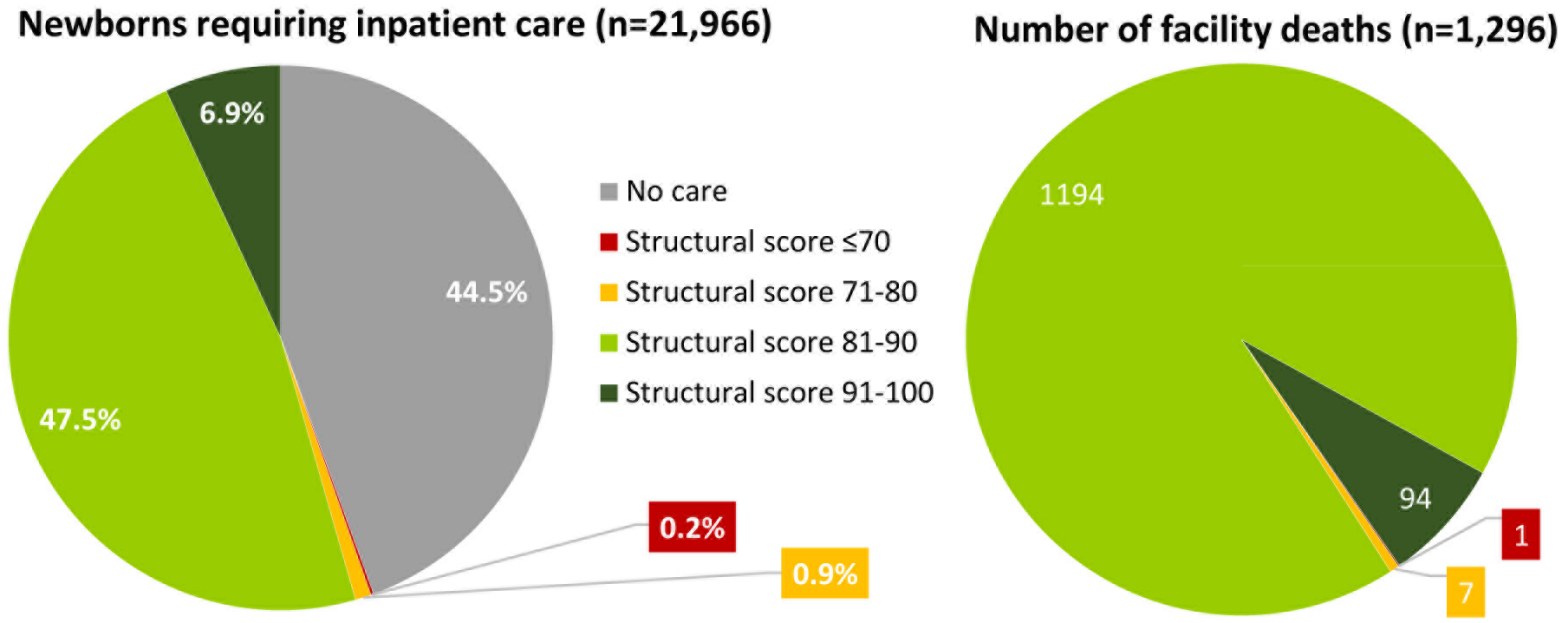
Levels of resource environment in which care is received by newborns requiring inpatient services and facility deaths occur in Nairobi City County.

Of the 1,296 deaths, 92% occurred in the 12/31 facilities with a structural score of 81–90% (Fig 5). Referral was reportedly more common from facilities with structural scores >90% (20% referral rate) and from those with structural scores ≤80% (32–34% referral rate) that were also often the smaller facilities (S2 Fig).

### Adequacy of neonatal care

Taken together, we suggest 21/31 of facilities providing INC were not able to offer an adequate level of care for common severe newborn illnesses due to inadequacies in structural capacity, or staffing or because they likely had too few admissions per year (<50) to sustain knowledge and skills. Nine (9/21; 78 admissions) of these facilities met the criteria of inadequacy for all three of these features. The 10/31 facilities (10,176 admissions) meeting minimal service criteria included four public, three mission, and three private sector facilities, though in the busiest of these 10 facilities considerable challenges to the provision of quality care still exist, particularly low nurse to patient ratios and overcrowding.

### Gaps in provision of care

When compared to the estimated total need for inpatient services for sick newborns in Nairobi City County (21,966 newborns, derived from Murphy et al [22]), 12,202 (56%, 12,202/21,966) accessed the INC facilities described in this report (Fig 5). Of these, 12.5% (1,520/12,202) and 85.5% (10,438/12,202) accessed facilities with structural scores 91–100% and 81–90% respectively, while 16.6% (2,026/12,202) accessed a facility we define as providing inadequate services.

## Discussion

Prior to this study, the Nairobi City County government, who assumed responsibility for healthcare for approximately four million people in 2013 following devolution, did not have a comprehensive understanding of neonatal services in the county, especially in the non-public sectors. This study provides a detailed landscape of the care being provided for small and sick newborns across Nairobi City County, highlighting important gaps in provision, access, and quality of care for this vulnerable population. Detailed data collection tools and protocols for neonatal service assessment have been developed during this study and made publicly available.[16]

We estimate that 44% of the expected small and sick newborns from the annual birth cohort in the county were likely not accessing care from the INC facilities we characterise, an additional approximately 9% of sick newborns access inadequate INC facilities. Additional findings support the suggestion that half of sick newborns do not access appropriate care–only an estimated 52% (63,953/122,632) of births in Nairobi City County during the study period occurred in one of the 32 facilities offering both maternity and INC (*unpublished data*). These findings suggest many women and their babies are receiving care from even less well-resourced facilities than those we describe and that many sick newborns (or mothers) may not be accessing the onward referral care they may need despite the high proportion of births taking place in facilities in Nairobi (88.7% [14]) overall. Of sick newborns accessing an INC facility, 71% are cared for in the public sector (4 of 34 facilities in Nairobi City County). Although these facilities achieved structural scores of 80–90% they are critically understaffed (4–15 patients per nurse) and overcrowded (bed occupancy >100% in 2 of 4 facilities).

It is important to acknowledge that structural quality does not always translate into quality care delivered to the patient. Resources must be utilised in line with clinical guidelines to provide safe and effective care, including appropriate temperature control, feeding regimes, hygiene (including hand washing), and avoidance of unnecessary invasive procedures. An example unearthed in this study relates to KMC. National guidelines in Kenya stating that all <2000 g birthweight newborns required admission for KMC. This study found 15% of admissions to be for newborns <2000 g and KMC wraps to be available in 22 of 31 facilities, with almost all facilities reporting to provide KMC services. However, discussion with our expert group suggests that few facilities are adequately implementing KMC. Most facilities do not have a dedicated KMC space and most facilities providing KMC are only doing so for a restricted number of hours per day. Further analysis of the process gaps of quality of care for neonatal patients in Nairobi County are published elsewhere (*Murphy et al*, *BMC Medicine*, *in press*).

**Fig 6:**
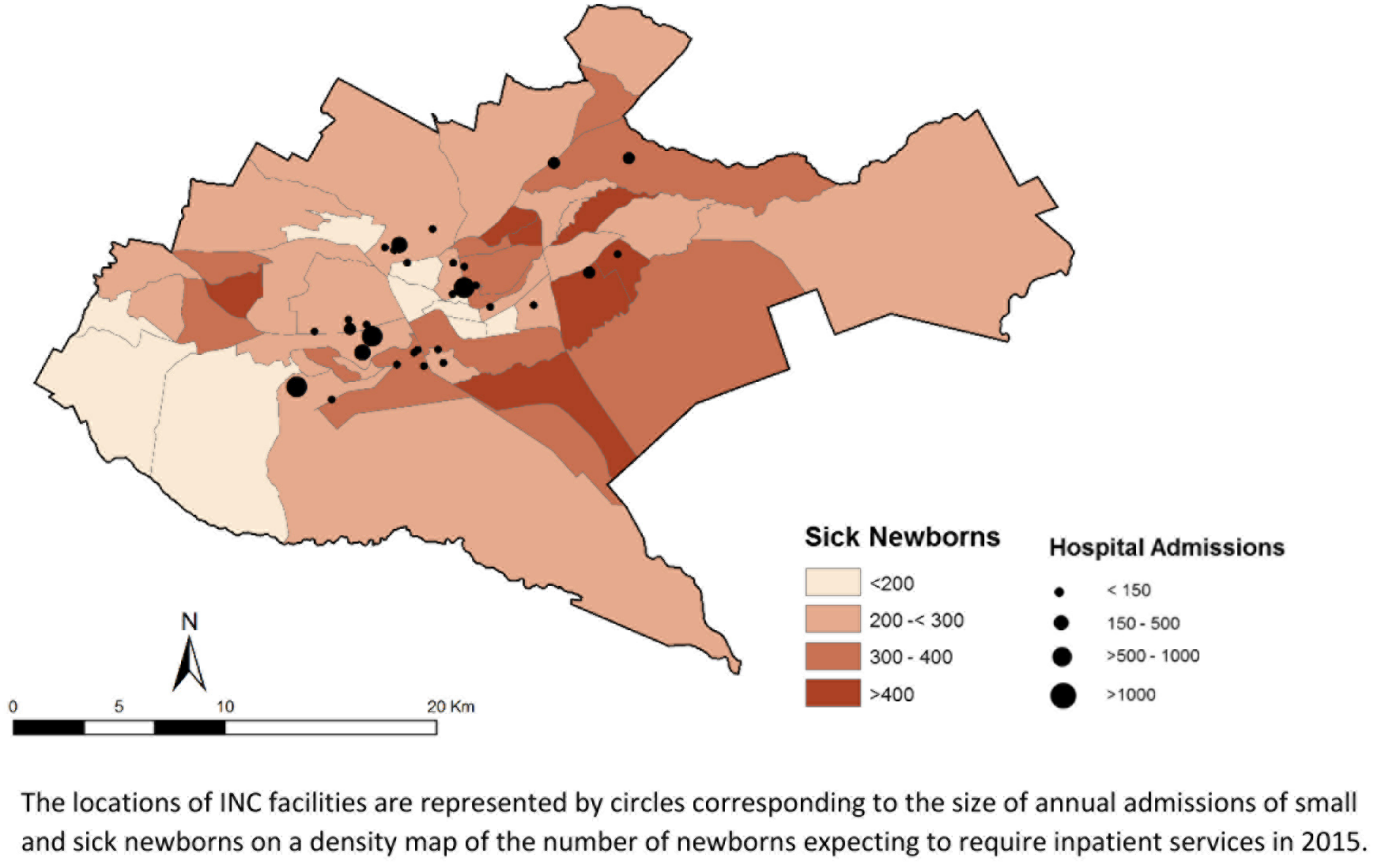
Map of sick newborn population and health facilities in Nairobi City County.

Providing adequate care for sick newborns requires specialised skills, equipment, and environment.[11, 26] Evidence from high-income countries suggests that expertise gained through specialisation linked to higher volumes of patients can improve outcomes, where structural capacity is adequate.[27–30] Currently, within Nairobi City County, 33 facilities (excluding a military-owned facility) appear to be offering inpatient neonatal care. Many of these facilities have very low/low occupancy. Although this suggests an ability to increase provision of neonatal care substantially, many of these INC facilities are very small, less well equipped, and often have few (if any) dedicated staff. An additional barrier to access is that most INC facilities in Nairobi typically charge substantial fees, which are unaffordable to the majority of Nairobi residents. Two thirds of the facilities had minimum daily NBU charges more than double (>30 USD) the 2017 minimum daily wage in Nairobi (6–14 USD). Thus, large parts of the population are currently excluded from accessing neonatal care due to: inadequate provision of health facilities and services; overcrowding and under-resourcing of the small number of adequately equipped facilities; and financial barriers. Physical barriers to healthcare may also exist given the current distribution of INC facilities, with large areas of predicted need for care being underserved in the county (Fig 6).

Our estimates suggest that there is a need to almost double neonatal services provision. Increasing access to smaller facilities through overcoming financial barriers (e.g. through insurance) may promote admissions to poorly resourced private sector facilities. By contrast, larger public facilities that largely offer free care would require substantial investment to ensure that an increase in patient numbers is met with an increase in physical resources and, especially, adequate nursing staff. Moreover, the county would benefit from a better planned and coordinated referral system to ensure that newborns are accessing care at the scale and quality needed.[31] Our findings show that referrals from one facility to another were more common from high cost, well-resourced facilities (often to the public sector when families could not pay) or from smaller facilities that have fewer medical staff, lower structural scores, and were less likely to have a dedicated NBU. Thus referral policies and plans need to be multi-sectoral.

It is recommended in countries such as the UK, even for babies who do not require intensive care, that there should be 1 nurse for every 2 to 4 sick babies[32, 33], with evidence suggesting higher mortality where such standards are not met.[32, 34, 35] In our setting, we found ratios as high as one nurse to 15 patients, with nursing numbers often being even lower during night shifts. Small facilities often had the appearance of better patient to nurse ratios, but these nurses were not dedicated staff as they also offer care during the same shift on other units. Such staff may not have specialised skills and/or experience in neonatal care.[28–30] Furthermore, these shared duties across wards means that sick newborns on these units may be left unattended by a nurse for extended periods of time (also suggested by informal observations of the study team). By contrast, larger facilities were found to have dedicated nursing staff but the highest patient to nurse ratios and mortality rates. The latter perhaps suggests a higher acuity of sick newborns also potentially resulting from the receipt of small and sick babies referred from smaller private facilities.

The problems of underuse of appropriate care and shortages in health workers can be compounded if resources, including health worker time and energy, are consumed by delivering unneeded care.[5, 36] Clear admission policies and organisation of care is, therefore, important to ensure care is provided in the most appropriate setting for sick newborns.[2] Such policies are, however, ill-defined in Kenya with admission policies to the facilities surveyed varying widely. Ten facilities admitted patients up to 28 days old to their NBU; 11 facilities reported having no age limit; and others reported age limits ranging from three days to one year old. All but one facility stated that they routinely admit low birth-weight newborns to their newborn unit but the definitions of low birthweight needing admission ranged from <1kg to 2.5kg. A third of facilities stated that they did not admit newborns born outside of their facility to the NBU. Furthermore, we suggest that 18% of admissions (2,605/14,807) to NBUs are inappropriate. The admission of such patients to the NBU varied substantially between facilities; in one facility, 37% of their admissions were for supportive care or minor conditions only. These newborns would be better cared for on post-natal wards with the mother. However, the broad problem of poor staffing, including post-natal wards, may have diverted these newborns to the newborn unit, detracting resources from those newborns in greatest need. There may also be perverse incentives for admission to the newborn unit in facilities levying charges for such admission.

Our study has both strengths and limitations. Our assessment of available equipment and drugs was conducted at one point of time. It is possible that the availability on that day was not a fair representation of the norm at a facility. Furthermore, we did not measure the quantity of resources available, with the exception of staff and beds and it is possible that available supplies do not match the needs of patients. We counted only cots and incubators that were specifically dedicated to the care of small and sick newborns in the NBU when calculating bed capacity. Some facilities commented that inpatient newborns were, on occasions, accommodated with the mother on the maternity ward. Nursing staff numbers may also vary with time, particularly in an environment where addition of temporary locum staff during busier periods is common. Admission diagnoses were captured from neonatal admission registers. We note that a different pattern of diagnosis was recorded in a random sample of medical records we obtained for the same study period (e.g. severe infection was recorded as an admission diagnosis for only 9.6% in registers but 18.9% in medical records). The discrepancy is likely due to admission registers being filled by nurses at the immediate point of admission, whereas the medical record is filled after medical assessment by a clinician. Residency data was not available for newborns. We found that 12.3% of women accessing the facilities we studied came from outside of Nairobi (unpublished data). We have no data on how many women or newborns might travel out of Nairobi for care. Further, while we describe referral patterns, individual babies could not be tracked and, thus, in some cases a baby may be counted as two admissions. Our estimate that 44% of sick newborns likely to need INC are likely not accessing it needs, therefore, to be interpreted with caution.

Previous studies in Kenya have also found shortcomings in the structural capacity of health facilities in Kenya to provide maternal, newborn, and child health.[8, 37–41] However, prior to this study, no comprehensive information was previously available on the availability or quality of inpatient neonatal health services in a highly urbanised setting across all sectors. This work was done with the support of an expert advisory stakeholder group, including partners from the Ministry of Health, Nursing Council of Kenya, University of Nairobi, and Nairobi City County.[22] Such an approach to stakeholder engagement aims to ensure that the outputs of the study are of direct relevance and use to local policy makers and practitioners who strive to improve the care for sick newborns and reduce neonatal mortality in this low-resource setting.

By 2030 in Kenya, more than 6000 newborn lives could be saved with childbirth and newborn care intervention packages alone[12], including basic inpatient care, such as that explored in this study. Information on the provision and quality of services will be crucial in order for governments to prioritise and plan service improvements to achieve such goals. The findings of this study have been communicated to policymakers and stakeholders, as well as individualised facility feedback being provided to each participating facility. The results have been well received and the need for and appreciation of data on both an individual facility level and a county level was clear. Ideally, local routine civil registration and vital statistics, health management information systems, and quality audit systems would be in place to inform governments on the needs, provision, and quality of care, respectively. In the meantime, our study provides valuable information to support evidence-based planning to strengthen healthcare for small and sick newborns.

## Supporting information

S1 Table Mean scores across domains. (DOCX)

S2 Table Number of facilities with availability of domain items. (DOCX)

S3 Table Number of facilities with availability equipment and drugs in the surgery theatre for maternity patients. (DOCX)

S4 Table Newborn patient characteristics (n = 12,202). (DOCX)

S1 Fig Distribution of structural quality across INC facilities by number of annual admissions. (DOCX)

S2 Fig Patient outcomes by level of care defined by structural score. (DOCX)

## Abbreviations

24/7: 24 hours a day for seven days a week
INC: inpatient neonatal care
LMICs: low- and middle-income countries
LOS: length of stay
mission: Private not-for-profit
NBUs: newborn units
private: private-for-profit

## References

[1] Countdown to 2015. A Decade of Tracking Progress for Maternal, Newborn and Child Survival: The 2015 Report. UNICEF and World Health Organisation, 2015.

[2] Moxon SG, Lawn JE, Dickson KE, Simen-Kapeu A, Gupta G, Deorari A, et al Inpatient care of small and sick newborns: a multi-country analysis of health system bottlenecks and potential solutions. BMC Pregnancy Childbirth. 2015;15 Suppl 2:S7 doi: 10.1186/1471-2393-15-S2-S7 26391335 10.1186/1471-2393-15-S2-S7 PMC4577807

[3] United Nations. Sustainable Development Goals: Goal 3 [cited 2017 26th May]. https://sustainabledevelopment.un.org/sdg3.

[4] Bhutta ZA, Das JK, Bahl R, Lawn JE, Salam Ra, Paul VK, et al Can available interventions end preventable deaths in mothers, newborn babies, and stillbirths, and at what cost? The Lancet. 2014;384:347–70. doi: 10.1016/S0140-6736(14)60792-3 . 24853604 10.1016/S0140-6736(14)60792-3

[5] Glasziou P, Straus S, Brownlee S, Trevena L, Dans L, Guyatt G, et al Evidence for underuse of effective medical services around the world. Lancet. 2017;390(10090):169–77. doi: 10.1016/S0140-6736(16)30946-1 . 28077232 10.1016/S0140-6736(16)30946-1

[6] Kruk ME, Kelley E, Syed SB, Tarp F, Addison T, Akachi Y. Measuring quality of health-care services: what is known and where are the gaps? Bull World Health Organ. 2017;95(6):389–A. doi: 10.2471/BLT.17.195099 28603302 10.2471/BLT.17.195099 PMC5463820

[7] Kruk ME, Pate M, Mullan Z. Introducing The Lancet Global Health Commission on High-Quality Health Systems in the SDG Era. Lancet Glob Health. 2017;5(5):e480–e1. doi: 10.1016/S2214-109X(17)30101-8 . 28302563 10.1016/S2214-109X(17)30101-8

[8] O'Neill K, Takane M, Sheffel A, Abou-Zahr C, Boerma T. Monitoring service delivery for universal health coverage: the Service Availability and Readiness Assessment. Bull World Health Organ. 2013;91(12):923–31. doi: 10.2471/BLT.12.116798 24347731 10.2471/BLT.12.116798 PMC3845262

[9] Hancioglu A, Arnold F. Measuring coverage in MNCH: tracking progress in health for women and children using DHS and MICS household surveys. PLoS Med. 2013;10(5):e1001391 doi: 10.1371/journal.pmed.1001391 23667333 10.1371/journal.pmed.1001391 PMC3646216

[10] Akachi Y, Kruk ME. Quality of care: measuring a neglected driver of improved health. Bull World Health Organ. 2017;95(6):465–72. doi: 10.2471/BLT.16.180190 28603313 10.2471/BLT.16.180190 PMC5463815

[11] Moxon SG, Ruysen H, Kerber KJ, Amouzou A, Fournier S, Grove J, et al Count every newborn; a measurement improvement roadmap for coverage data. BMC Pregnancy Childbirth. 2015;15 Suppl 2:S8 doi: 10.1186/1471-2393-15-S2-S8 26391444 10.1186/1471-2393-15-S2-S8 PMC4577758

[12] Keats EC, Ngugi A, Macharia W, Akseer N, Khaemba EN, Bhatti Z, et al Progress and priorities for reproductive, maternal, newborn, and child health in Kenya: a Countdown to 2015 country case study. Lancet Glob Health. 2017;5(8):e782–e95. doi: 10.1016/S2214-109X(17)30246-2 . 28716350 10.1016/S2214-109X(17)30246-2 PMC5599303

[13] African Population and Health Research Center (APHRC). Population and Health Dynamics in Nairobi’s Informal Settlements: Report of the Nairobi Cross-sectional Slums Survey (NCSS) 2012. Nairobi: APHRC, 2014.

[14] Kenya National Bureau of Statistics. 2014 Kenya Demographic and Health Survey (2014 KDHS). Nairobi, Kenya: Kenya National Bureau of Statistics, 2015.

[15] Murphy GA, Gathara D, Aluvaala J, Mwachiro J, Abuya N, Ouma P, et al Nairobi Newborn Study: a protocol for an observational study to estimate the gaps in provision and quality of inpatient newborn care in Nairobi City County, Kenya. BMJ Open. 2016;6(12):e012448 doi: 10.1136/bmjopen-2016-012448 28003285 10.1136/bmjopen-2016-012448 PMC5223685

[16] The Global Health Network. Estimating the gaps in provision and quality of inpatient newborn care. https://globalresearchmethods.tghn.org/methodology-projects/estimating-gaps-provision-and-quality-inpatient-newborn-care/.

[17] Ministry of Medical Services, Ministry of Public Health and Sanitation. Master Facility List Kenya: Division of Health Information Systems, Department of Standards and Regulatory Services; 2010. http://kmhfl.health.go.ke/#/home.

[18] Noor AM, Alegana VA, Gething PW, Snow RW. A spatial national health facility database for public health sector planning in Kenya in 2008. Int J Health Geogr. 2009;8:13 doi: 10.1186/1476-072X-8-13 19267903 10.1186/1476-072X-8-13 PMC2666649

[19] Harris PA, Taylor R, Thielke R, Payne J, Gonzalez N, Conde JG. Research electronic data capture (REDCap)—a metadata-driven methodology and workflow process for providing translational research informatics support. J Biomed Inform. 2009;42(2):377–81. doi: 10.1016/j.jbi.2008.08.010 18929686 10.1016/j.jbi.2008.08.010 PMC2700030

[20] Kenya National Bureau of Statistics. 2009 Kenya Population and Housing Census. Nairobi, Kenya: Kenya National Bureau of Statistics, 2009.

[21] Kenya National Bureau of Statistics. The 1999 Population and Housing Census. Kenya National Bureau of Statistics, 2001 January 2001. Report No.

[22] Murphy GAV, Waters D, Ouma PO, Gathara D, Shepperd S, Snow RW, et al Estimating the need for inpatient neonatal services: an iterative approach employing evidence and expert consensus to guide local policy in Kenya. BMJ Glob Health. 2017;2(4):e000472 doi: 10.1136/bmjgh-2017-000472 29177099 10.1136/bmjgh-2017-000472 PMC5687539

[23] EasyCalculation. How to Calculate Inpatient Bed Occupancy Rate in Hospital [cited 2017 9th July]. www.easycalculation.com/medical/learn-inpatient-occupancy-rate.php.

[24] District health information software 2 (DHIS2) [cited 2015 9th December]. hiskenya.org.

[25] Kazungu JS, Barasa EW. Examining levels, distribution and correlates of health insurance coverage in Kenya. Trop Med Int Health. 2017;22(9):1175–85. doi: 10.1111/tmi.12912 . 28627085 10.1111/tmi.12912 PMC5599961

[26] Lawn JE, Blencowe H, Oza S, You D, Lee AC, Waiswa P, et al Every Newborn: progress, priorities, and potential beyond survival. Lancet. 2014;384(9938):189–205. doi: 10.1016/S0140-6736(14)60496-7 . 24853593 10.1016/S0140-6736(14)60496-7

[27] Horbar JD. The Vermont Oxford Network: evidence-based quality improvement for neonatology. Pediatrics. 1999;103(1 Suppl E):350–9. . 9917477

[28] Hamilton KE, Redshaw ME, Tarnow-Mordi W. Nurse staffing in relation to risk-adjusted mortality in neonatal care. Arch Dis Child Fetal Neonatal Ed. 2007;92(2):F99–F103. doi: 10.1136/adc.2006.102988 17088341 10.1136/adc.2006.102988 PMC2675478

[29] Phibbs CS, Bronstein JM, Buxton E, Phibbs RH. The effects of patient volume and level of care at the hospital of birth on neonatal mortality. JAMA. 1996;276(13):1054–9. . 8847767

[30] Needleman J, Buerhaus P, Mattke S, Stewart M, Zelevinsky K. Nurse-staffing levels and the quality of care in hospitals. N Engl J Med. 2002;346(22):1715–22. doi: 10.1056/NEJMsa012247 . 12037152 10.1056/NEJMsa012247

[31] Kenyan Ministry of Health Division of Emergency and Disaster Risk Management. Kenya Health Sector Referral Strategy (2014–2018). Nairobi, Kenya: 2014.

[32] British Association of Perinatal Medicine. Standards For Hospitals Providing Neonatal Intensive And High Dependency Care (Second Edition)2001.

[33] National Association of Neonatal Nurses (USA). Position Statement #3009—Minimum RN Staffing in NICUs.2009.

[34] Sherenian M, Profit J, Schmidt B, Suh S, Xiao R, Zupancic JA, et al Nurse-to-patient ratios and neonatal outcomes: a brief systematic review. Neonatology. 2013;104(3):179–83. doi: 10.1159/000353458 . 23941740 10.1159/000353458

[35] Watson SI, Arulampalam W, Petrou S, Marlow N, Morgan AS, Draper ES, et al The effects of a one-to-one nurse-to-patient ratio on the mortality rate in neonatal intensive care: a retrospective, longitudinal, population-based study. Arch Dis Child Fetal Neonatal Ed. 2016;101(3):F195–200. doi: 10.1136/archdischild-2015-309435 . 26860480 10.1136/archdischild-2015-309435

[36] Saini V, Garcia-Armesto S, Klemperer D, Paris V, Elshaug AG, Brownlee S, et al Drivers of poor medical care. Lancet. 2017;390(10090):178–90. doi: 10.1016/S0140-6736(16)30947-3 . 28077235 10.1016/S0140-6736(16)30947-3

[37] Kruk ME, Chukwuma A, Mbaruku G, Leslie HH. Variation in quality of primary-care services in Kenya, Malawi, Namibia, Rwanda, Senegal, Uganda and the United Republic of Tanzania. Bull World Health Organ. 2017;95(6):408–18. doi: 10.2471/BLT.16.175869 28603307 10.2471/BLT.16.175869 PMC5463807

[38] MEASURE Evaluation PIMA. Health Facility Readiness to Provide Emergency Obstetric and Newborn Care in Kenya: Results of a 2014 Assessment of 13 Kenyan Counties with High Maternal Mortality. Nairobi, Kenya: MEASURE Evaluation PIMA, University of North Carolina at Chapel Hill, 2016.

[39] Aluvaala J, Nyamai R, Were F, Wasunna A, Kosgei R, Karumbi J, et al Assessment of neonatal care in clinical training facilities in Kenya. Archives of disease in childhood. 2015;100:42–7. doi: 10.1136/archdischild-2014-306423 . 25138104 10.1136/archdischild-2014-306423 PMC4283661

[40] Gathara D, Opiyo N, Wagai J, Ntoburi S, Ayieko P, Opondo C, et al Quality of hospital care for sick newborns and severely malnourished children in Kenya: a two-year descriptive study in 8 hospitals. BMC Health Serv Res. 2011;11:307 doi: 10.1186/1472-6963-11-307 22078071 10.1186/1472-6963-11-307 PMC3236590

[41] Opondo C, Ntoburi S, Wagai J, Wafula J, Wasunna A, Were F, et al Are hospitals prepared to support newborn survival?—An evaluation of eight first-referral level hospitals in Kenya. Trop Med Int Health. 2009;14(10):1165–72. doi: 10.1111/j.1365-3156.2009.02358.x 19695001 10.1111/j.1365-3156.2009.02358.x PMC2751740

