# Supplementary material for: What capacity exists to provide essential inpatient care to small and sick newborns in a high mortality urban setting? - A cross-sectional study in Nairobi City County, Kenya
Source: PLoS One. 2018 Apr 27;13(4):e0196585. doi: 10.1371/journal.pone.0196585 (PMC5922525; doi:10.1371/journal.pone.0196585)
Supplement: S1 Table — (DOCX) [file pone.0196585.s001.docx]

**Appendix Table S1: Mean scores across domains**

| **Domain (n=number of items)** | **Mean (range) percentile score out of 100** | | | |
| --- | --- | --- | --- | --- |
|  | **Total** | **Public (n=4)** | **Mission (n=6)** | **Private (n=21)** |
| Infrastructure (n=3) | 98.9 (66.7-100) | 91.7 (66.7-100) | 100 (100-100) | 100 (100-100) |
| Laboratory tests (n=9) | 83.2 (0-100) | 75.0 (44.4-100) | 87 (66.7-100) | 83.6 (0-100) |
| Hygiene (n=14) | 93.5 (71.4-100) | 92.9 (78.6-100) | 94 (78.6-100) | 93.5 (71.4-100) |
| Safe delivery equipment and drugs for mothers (n=37) | 67.8 (27-89.2) | 80.4 (64.9-89.2) | 78.8 (67.6-89.2) | 62 (27.0-83.8) |
| Resuscitation equipment on delivery ward (n=20) | 90.3 (50-100) | 87.5 (75.0-95.0) | 96.7 (95.0-100) | 89 (50.0-100) |
| Essential ward equipment in the NBU for treatment and diagnostic procedures (n=18) | 86.6 (11.1-100) | 94.4 (88.9-100) | 91.7 (83.3-100) | 83.6 (11.1-100) |
| IV fluid and feeds in the NBU (n=8) | 80.6 (0-100) | 90.6 (87.5-100) | 89.6 (75.0-100) | 76.2 (0-100) |
| NBU drugs (n=17) | 81.8 (17.6-100) | 77.9 (70.6-94.1) | 88.2 (76.5-100) | 80.7 (17.6-100) |

** For the 1/31 facility that did not provide maternity services, the numerator and denominator were adjusted to exclude delivery ward and maternity items.*
