# Supplementary material for: What capacity exists to provide essential inpatient care to small and sick newborns in a high mortality urban setting? - A cross-sectional study in Nairobi City County, Kenya
Source: PLoS One. 2018 Apr 27;13(4):e0196585. doi: 10.1371/journal.pone.0196585 (PMC5922525; doi:10.1371/journal.pone.0196585)
Supplement: S2 Table — (DOCX) [file pone.0196585.s002.docx]

**Appendix Table S2: Number of facilities with availability of domain items**

| **Infrastructure** | **Facility (n=31)** |
| --- | --- |
| Consistent power (outages <monthly) or generator serving NBU | 30 |
| Heating in NBU | 31 |
| Running water | 31 |

| **Laboratory tests** | **Total (n=31)** |
| --- | --- |
| **MINIMUM PACKAGE** |  |
| Open 24/7 | 29 |
| Test for haemoglobin | 30 |
| Test for bilirubin (blood test) | 28 |
| Glucose tests | 31 |
| Blood grouping and cross match | 30 |
| Electrolytes (sodium / potassium) | 28 |
| **All minimum package*** | **27** |
| **ADDITIONAL DRUGS** |  |
| Blood bank | 30 |
| Blood slide microscopy for malaria parasites | 30 |
| Test for direct Coombs test | 29 |
| Urea or creatinine | 28 |
| Liver function tests (enzymes e.g. AST/ALT) | 28 |
| Microscopy & culture: Pus swab and urine culture | 27 |
| CSF microscopy | 21 |
| Coagulation profile | 21 |
| Blood culture ability | 18 |

** Minimum requirement to score above zero*

| **Basic hygiene and safety equipment** | **Delivery ward (n=30*)** | **Newborn Unit (n=31)** |
| --- | --- | --- |
| Cleaning/disinfectant supplies | 30 | 31 |
| Sharps disposed in a special container | 30 | 30 |
| Clean gloves available | 30 | 30 |
| Separate clinical and non-clinical waste | 30 | 27 |
| Sinks with soap and water for hand-washing | 29 | 27 |
| Mother has access to running water | 30 | 27 |
| Alcohol hand rub | 23 | 25 |

**One of the 31 facilities was a children’s hospital and does not provide maternity services*

| **Safe delivery equipment and drugs for mothers** | **Delivery ward (n=30*)** |
| --- | --- |
| **EQUIPMENT** |  |
| Thermometer | 20 |
| Sterile syringes | 29 |
| Sterile needles | 29 |
| Sterile vaginal examination packs | 30 |
| Sterile delivery set (complete) | 30 |
| A stethoscope | 21 |
| Amnicots/sterile Kocker’s forceps for artificial rupture of membrane | 28 |
| Urine dipstick kits/strips | 13 |
| Urinary catheters | 26 |
| Vacuum (such as Kiwi) for assisted vaginal delivery | 19 |
| Manual vacuum aspirator (MVA) | 11 |
| Long gloves for manual removal of placenta | 12 |
| Guedel airways – these should be a full range of sizes | 17 |
| Bag Valve Mask (BVM) device: adult size bag and mask | 27 |
| Oxygen source (any and working) | 30 |
| Nasal catheters/prongs | 26 |
| Oxygen face –masks (with and without reservoir bags) | 29 |
| Oxygen flow regulators | 28 |
| Laryngoscope | 15 |
| Laryngoscope blades (straight, curved, and different sizes) | 16 |
| Endotracheal tubes (of different sizes) | 16 |
| IV fluid giving sets | 29 |
| Blood giving set | 26 |
| Adult IV cannulae | 30 |
| Blood pressure monitor (any and working) | 27 |
| **DRUGS AVAILABLE ON THE WARD** |  |
| Adrenaline | 25 |
| Magnesium sulphate | 26 |
| Lasix | 18 |
| Digoxin | 3 |
| Morphine | 6 |
| Oxytocin | 24 |
| Dexamethasone | 16 |
| Prostaglandin F2 alpha | 10 |
| Calcium gluconate | 24 |
| Penicillin | 6 |
| Gentamicin | 7 |
| Ceftriaxone/Cefuroxime | 4 |

**One of the 31 facilities was a children’s hospital and does not provide maternity services*

| **Resuscitation equipment on delivery ward** | **Delivery ward (n=30*)** |
| --- | --- |
| Thermometer | 19 |
| Weighing scales | 26 |
| Sterile syringes | 29 |
| Sterile needles | 28 |
| Warm dry towels for dying and wrapping the newborn | 27 |
| Sterile cord clamp | 29 |
| Sterile scissors | 27 |
| A firm stable surface for placing the newborn for resuscitation (where warmth can be maintained) | 30 |
| An overhead light source above the surface for resuscitation | 28 |
| A clock in view or reach of surface for resuscitation | 28 |
| A stethoscope | 25 |
| Suction tubes/catheters | 30 |
| Suction Machine | 28 |
| Guedel airways – these should be a full range of sizes | 16 |
| Bag Valve Mask (BVM) devices: bag size 500 ml or750 ml, that are in working order with newborn face masks (sizes 0 and 1) | 27 |
| Oxygen source (any and working) | 28 |
| Nasal catheters/prongs | 25 |
| Oxygen face –masks (with and without reservoir bags) | 26 |
| Oxygen flow regulators | 27 |
| Warming equipment-working radiant heaters | 28 |

**One of the 31 facilities was a children’s hospital and does not provide maternity services*

| **Essential ward equipment in the NBU for treatment and diagnostic procedures** | **Newborn Unit (n=31)** |
| --- | --- |
| Thermometer | 26 |
| Weighing scales | 30 |
| Sterile syringes | 30 |
| Sterile needles | 29 |
| A stethoscope | 27 |
| Suction tubes/catheters | 26 |
| Suction Machine | 26 |
| Guedel airways – these should be a full range of sizes | 19 |
| Bag Valve Mask (BVM) devices: bag size 500 ml or750 ml, that are in working order with newborn face masks (sizes 0 and 1) | 28 |
| Oxygen source (any and working) | 30 |
| Nasal catheters/prongs | 27 |
| Oxygen face –masks (with and without reservoir bags) | 27 |
| Oxygen flow regulators | 30 |
| Warming equipment-working radiant heaters | 30 |
| Kangaroo mother care wraps | 22 |
| Phototherapy equipment | 30 |
| Eye protection for phototherapy | 26 |
| Blood transfusion giving set | 20 |

| **IV fluid and feeds in the NBU** | **Newborn Unit (n=31)** |
| --- | --- |
| Feeding cups for giving expressed breast milk | 23 |
| IV fluid burette | 25 |
| Infusion set / adult IV fluid set | 25 |
| Paediatric cannulae | 28 |
| Nasogastric tube (FG6 or 8 or other) | 27 |
| glucose 10% | 30 |
| normal saline IV or ringers lactate | 29 |
| term formula | 19 |

| **NBU drugs** | **Newborn Unit (n=31)** |
| --- | --- |
| Vitamin K | 17 |
| Nevirapine solution | 15 |
| Prophylactic tetracycline eye ointment* | 25 |
| Phenobarbitone (injection) | 19 |
| Phenytoin (injection) * | 27 |
| Aminophyline* | 30 |
| Penicillin (injection) | 14 |
| Gentamicin or Amikacin | 30 |
| Ampicillin / Cloxacillin (injection) * | 29 |
| Oral Cloxacillin * | 27 |
| Oral erythromycin* | 29 |
| Metronidazole (injection) * | 30 |
| Ceftriaxone or cefotaxime* | 30 |
| Ferrous Fumarate suspension* | 27 |
| Folate drops* | 27 |
| Multivitamin syrup/drops* | 29 |
| Intravenous (Anti-D) immunoglobulin (for rhesus disease) * | 26 |

** Considered available if on the ward or in a store*
