# Supplementary material for: What capacity exists to provide essential inpatient care to small and sick newborns in a high mortality urban setting? - A cross-sectional study in Nairobi City County, Kenya
Source: PLoS One. 2018 Apr 27;13(4):e0196585. doi: 10.1371/journal.pone.0196585 (PMC5922525; doi:10.1371/journal.pone.0196585)
Supplement: S3 Table — (DOCX) [file pone.0196585.s003.docx]

**Appendix Table S3: Number of facilities with availability equipment and drugs in the surgery theatre for maternity patients**

| **Maternity patient surgery theatre** | **Surgery theatre (n=30*)** |
| --- | --- |
| Designated scrubbing area | 30 |
| Functional operating lights | 30 |
| Oxygen supply | 30 |
| Standard operating table | 30 |
| Functional anaesthetic machine | 30 |
| Suction machine | 30 |
| Oropharyngeal airways | 30 |
| Endotracheal tubes | 30 |
| Laryngoscopes | 30 |
| Sterile needles | 30 |
| Sterile spinal needles | 30 |
| IV cannulae | 30 |
| Adult size Ambu bag and mask | 30 |
| Diathermy machine | 29 |
| Caesarian section sets (complete) | 29 |
| Dilatation and curettage set (complete) | 29 |
| Designated recovery area | 28 |
| Resuscitaire or firm surface with heater for receiving neonates | 28 |
| Hysterectomy set (complete) | 28 |
| Manual vacuum aspiration kit (complete) | 28 |
| **DRUGS AVAILABLE IN THE THEATRE** | |
| Atropine sulphate | 30 |
| Lignocaine | 30 |
| Adrenaline | 30 |
| Nitrous Oxide | 30 |
| Normal saline | 30 |
| 5% Dextrose | 30 |
| 50% Dextrose | 30 |
| Diazepam | 29 |
| Ketamine | 29 |
| Propofol | 29 |
| Halothane | 29 |
| Bupivacaine | 29 |
| Ringer’s lactate | 29 |
| Suxamethonium | 28 |
| Mercaine | 28 |
| Oxytocin | 28 |
| Hyoscine hydrobromide | 27 |
| Misoprostol | 27 |
| Promethazine | 26 |
| Thiopentone Sodium | 25 |
| Morphine | 23 |
| Pancuronium | 22 |

**One of the 31 facilities was a children’s hospital and does not provide maternity services*
