# Supplementary material for: What capacity exists to provide essential inpatient care to small and sick newborns in a high mortality urban setting? - A cross-sectional study in Nairobi City County, Kenya
Source: PLoS One. 2018 Apr 27;13(4):e0196585. doi: 10.1371/journal.pone.0196585 (PMC5922525; doi:10.1371/journal.pone.0196585)
Supplement: S4 Table — (DOCX) [file pone.0196585.s004.docx]

**Appendix Table S4:** Newborn patient characteristics (n=12,202)

| Patient characteristics | | % |
| --- | --- | --- |
| Received from | Inborn | 14.4 |
|  | Referred | 2.3 |
|  | From home | 0.2 |
|  | Not recorded | 83.1 |
| Sex | Male | 48.9 |
|  | Female | 43.9 |
|  | Not recorded | 7.2 |
| Age at admission | <24 hours | 49.3 |
|  | 1-2 days | 20.1 |
|  | 3-7 days | 6.5 |
|  | 1-4 weeks | 2.3 |
|  | Not recorded | 21.8 |
| Gestational age | Very preterm | 1.2 |
|  | Moderate/late preterm (32-36 weeks) | 2.4 |
|  | Term (37-42 weeks) | 5.4 |
|  | Over-term (>42 weeks) | 0.1 |
|  | Not recorded | 91.0 |
| Birth weight | <1kg | 1.8 |
|  | 1-<1.5kg | 4.3 |
|  | 1.5-<2.0 Kg | 8.8 |
|  | 2.0-<2.5 Kg | 12.4 |
|  | 2.5-<4 Kg | 44.5 |
|  | >=4 Kg | 3.9 |
|  | Not recorded | 24.4 |
