# Supplementary material for: What capacity exists to provide essential inpatient care to small and sick newborns in a high mortality urban setting? - A cross-sectional study in Nairobi City County, Kenya
Source: PLoS One. 2018 Apr 27;13(4):e0196585. doi: 10.1371/journal.pone.0196585 (PMC5922525; doi:10.1371/journal.pone.0196585)
Supplement: S2 Fig — (DOCX) [file pone.0196585.s006.docx]

**Appendix Fig S2:** Patient outcomes by level of care defined by structural score
